# Narrative Review of Electronic Health Record Systems in Anesthesia: Benefits, Risks, and Medico-Legal Considerations in the United States of America

**DOI:** 10.1007/s10916-025-02221-z

**Published:** 2025-06-23

**Authors:** George Tewfik, Beth Minzter, Franklin Chiao, Joel Zivot, Matthew Wecksell, Allan F. Simpao, CIIT Collaborators, Vikas O’Reilly-Shah, Kent Berg, Ellen Wang, Jonathan M. Tan, Kristin Ondecko, Mark Banoub, Michael Pesce, Reem Khatib, Ramon E. Abola

**Affiliations:** 1 Rutgers New Jersey Medical School, Newark, NJ USA; 2 Cleveland Clinic, Cleveland, OH USA; 3 Westchester Medical Center, New York Medical College, Valhalla, NY USA; 4 Emory School of Medicine, Atlanta, GA USA; 5 Children’s Hospital of Philadelphia, University of Pennsylvania Perelman School of Medicine, Philadelphia, PA USA

## Abstract

Electronic health records (EHRs) have transformed healthcare delivery and documentation by accurately capturing routine care and critical events through automated data recording. EHRs also enable clinical decision support, quality improvement initiatives, and large-scale research. A narrative review has been constructed using relevant research regarding medicolegal liability associated with EHRs, related to anesthesia care. EHRs have created new liability exposures through alert fatigue, system errors, and inappropriate use of functions such as copy-and-paste. Ethical issues and concerns with EHRs include privacy, informed consent, and secondary data uses. Metadata, “data about the data”, provides insight into record authenticity, clinician involvement in care, and communication between providers. However, EHR metadata is legally discoverable, and courts have compelled its release to plaintiffs despite hospital objections. This narrative review covers the benefits of EHRs in anesthesiology practice, discusses medicolegal liability and ethical concerns, and highlights a method for assessing medicolegal risks using the EHR.

**Supplementary Information:**

The online version contains supplementary material available at 10.1007/s10916-025-02221-z.

## Historical Context of Electronic Health Records (EHRs) in Anesthesiology

Anesthesiology has long been at the forefront of technological advancements aimed at enhancing patient safety. Anesthesia information management systems (AIMS) were developed in the 1980 s and 1990 s for intraoperative record keeping, either as replacement for, or a complement to, paper records [1]. In the 2010 s, EHRs frequently began to feature integrated AIMS functionality, which includes the ability to document perioperative notes, administration of medications, fluids and blood products, data from intraoperative records drawn from vital sign monitors and anesthesia workstations, as well as documentation for billing and regulatory compliance [2, 3]. Enhanced clinical documentation and automated vital sign data entry have created unbiased and precise records, which offer augmented transparency, legal integrity and protection [3]. 

## Clinical and Operational Benefits of EHRS in Anesthesia Practice

In anesthesiology, particularly in the United States, the use of EHR not only facilitates perioperative record-keeping, but also provides voluminous data that supports clinical decision-making, enhances research capabilities, and provides an avenue for quality improvement [4]. Organizations such as the Multicenter Perioperative Outcomes Group (MPOG) utilize EHR data across dozens of clinical sites to support local research, quality, and educational initiatives [5].

**Fig. 1:**
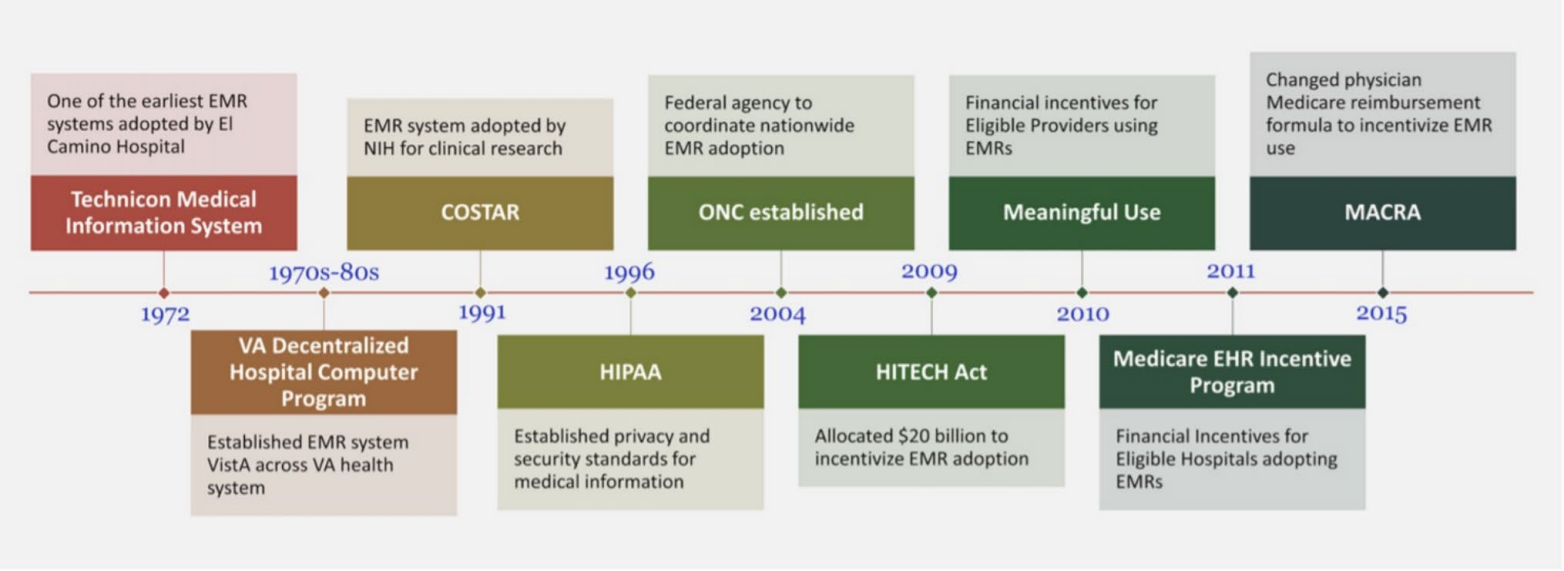
Summary of major federal legislation and programs promoting EHR adoption

The accuracy of data capture during routine perioperative care is significantly improved by EHR and AIMS which allow for rapid organization and retrieval of pertinent information (See Fig. 1 for brief timeline of EHR implementation in the US). The capture of real-time physiologic data provides immediate and precise documentation of patient vital signs, ventilator data, and data from other monitors, allowing for a greater focus on patient care. Immediate recording reduces the risk of both inadvertent and intentional errors in documentation, allows for more granular capture of data, and may negate the potential for misinterpretation linked with illegible handwriting—a common issue with traditional paper-based systems [6]. These benefits become particularly apparent during acute perioperative events, which often necessitate rapid and judicious decision-making under significant cognitive load [7].

AIMS that are integrated with the hospital’s EHR system offer additional safety benefits. By using a single medication administration record, these systems can lower the risk of missed administration, or risk of overdose, for crucial medications [8, 9]. EHRs also enable enhanced visualization of data in anesthesiology practice, facilitating use of data to track perioperative practice patterns and improve quality [10, 11]. In addition, EHR data can be used to create individualized, data-driven feedback on anesthesia care, such as electronic dashboards to improve anesthesiology residents’ documentation of neuromuscular block levels [12].

EHRs provide anesthesiologists with key information in the perioperative period, such as familial history of malignant hyperthermia, drug-drug interactions, and allergies.EHRs serve as a foundation for Clinical Decision Support (CDS) systems, using patient-specific information to guide evidence-based care. For example, EHR alerts can warn anesthesiologists of potentially difficult intubations [13], or to prompt antibiotic redosing. A study examining an EHR intervention of antibiotic redosing saw adherence improved from 4.3% to 73% simply because of improving tracking and alerts in an institution’s EHR [14]. Information transfer during perioperative care may also be enhanced via EHR use. Integration of a postanesthesia care unit (PACU) handoff checklist, for example, significantly increased the percentage of accurate information provided to PACU personnel, while not considerably affecting the duration of handoffs [15].

EHR systems have facilitated significant, impactful research in anesthesiology and brought a paradigm shift in the operation of clinical trials. By providing a panoramic view of patient characteristics and clinical histories, EHR assists in identifying suitable candidates for clinical trials, making the recruitment process more efficient. EHRs can serve as a valuable resource for health services research in anesthesiology, such as evaluating the impact of perioperative management strategies on healthcare utilization and costs. In addition, EHR may be able to integrate even more data regarding activities of the anesthesia clinician using “automated detection of anesthetic activity” [16]. This technology combines use of radiofrequency identification technology with received signal strength measurements and self-organizing maps to determine anesthetists'location, orientation, and stance [16]. Task analysis may be integrated as data into EHR for both quality and research purposes in the future.

Efficiently sharing vital patient information across diverse health systems to patients and institutions can eliminate redundant testing and heighten awareness of patients’ health conditions. The digital and uniform format of EHRs may be strengthened by interoperability standards like Health Level Seven International's (HL7) Fast Healthcare Interoperability Resources (FHIR), built on standardized vocabularies such as SNOMED CT (Systematized Nomenclature of Medicine – Clinical Terms) and LOINC (Logical Observation Identifiers Names and Codes) [17].

EHRs also serve as a critical foundation for anesthesiology practices and health systems to conduct and implement patient safety and quality improvement initiatives. For example, EHR data have been leveraged to improve surgical scheduling and efficiency in time management [18]. The transition to EHRs has enabled increased data availability and expanded opportunities for quality measurement [19]. 

Machine learning and artificial intelligence (AI) have been increasingly applied to EHR data to improve perioperative care [20]. For example, AI-driven CDS can forecast the likelihood of postoperative nausea and vomiting based on patient, anesthetic, and surgical factors, offering guidance on, and improving adherence to, prophylaxis [20]. The development of more complex algorithms, like those predicting intraoperative hypotension, presents an exciting glimpse into future possibilities [21]. The introduction of high-caliber large language models (LLMs) promises another revolution in patient care and the quality of delivery [22]. These are all relatively new concepts that will evolve the landscape of liability issues as people use more AI applications in the perioperative setting.

## Medicolegal Risks Associated with EHR Use

### Legal Frameworks and Medical Negligence Principles

EHRs are a critical consideration when discussing medicolegal liability. Medical malpractice has some historical considerations and principles. According to Black’s Law Dictionary, “common law” is defined as the body of law derived from judicial decisions, rather than from statutes or constitutions [23]. Another word for common law is case law and one third of the world’s population utilizes the common law as a legal system or incorporates the common law in a mixed system that combines the common law with civil law. Medical practice classically runs afoul of the common law in matters of medical negligence. Within the common law, negligence has four elements: 1) duty, 2) breach, 3) causation, and 4) damages. Practically, negligence occurs when physician practice departs from the standard of care and consequently a material harm ensues. This is known as a tort which is a civil wrong other than a breach of contract. The remedy of a tort wrong is restitution by converting damages to a dollar amount. The tortfeasor (the person that commits the tort) must pay the damages.

The legal structure of modern medical negligence draws from Donoghue v. Stevenson the landmark House of Lords court decision in Scots delict law and English tort law [24–26]. *Donoghue* set the standard for medical negligence worldwide in every legal jurisdiction that uses the common law. The case held that a manufacturer held a duty of care which was breached because it was reasonably foreseeable that a failure to ensure safety would lead to a harm to a consumer. Medical negligence requires that by action or inaction, a harm is reasonably foreseeable.

### System and User-Related EHR Errors

While EHR systems offer many benefits to clinical practice and research in anesthesiology, EHRs create new and different physician and hospital liability exposures. In fact, EHR-related lawsuits have increased in recent years as growth in use has occurred. Approximately two-thirds of EHR-related claims involved user-related factors and 40–60% involved system factors, with some cases involving more than one contributing factor according to analysis of claims in the CRICO Comparative Benchmark System (CBS) [27, 28]. Graber et al. investigated malpractice claims for a two-year period using the CRICO Comparative Benchmark System (CBS) and found 248 cases in which EHR-related events were cited as contributing factors in the legal action [28]. In those 248 cases, only 7 were identified in which anesthesiology was the primary medical service. Cases were also found in ambulatory (59%), inpatient (31%), and emergency (10%) settings, often involving substantial harm or death [28]. Thirty percent of the errors were medication related, 30% were errors related to diagnosis, and 30% were related to medical/surgical treatment [28]. 

This study differentiated EHR-related contributing factors between those related to systems and those caused by the user. System factors refer to the design, technology, and security of the system whereas user-related factors relate to the person using the system such as inaccurate patient information, entering the wrong medication, and copy and paste mistakes. Systems related issues included such components as software design, routing of data, systemic malfunction, incompatible systems and failures of alerts or decision support. User-related errors included incorrect information, pre-populated forms and poor training or education. The highest individual source of errors in malpractice cases in which EHR was listed as a contributing factor was attributed to medication discrepancies (31%), including ordering, dispensing or administration errors [23–26]. Other issues cited in litigation surrounding EHR use include alert fatigue, workarounds, and insufficient use of training and education. Some sources of errors include drop-down menus, which may omit less frequently encountered scenarios [29]. Suboptimal CDS design contributes to high override rates and alert fatigue, which may affect liability [30]. Excessive medication interaction alerts can appear despite negligible potential risk; legal implications remain ambiguous if CDS alerts are removed or if clinicians do not adhere to these alerts [30]. Auto-correction and auto-population in text entry and documentation can input incorrect or outdated information, which is the leading cause for EHR-related malpractice claims [29]. 

Clinicians often have access to huge amounts of patient data; however, there is no current statute or precedent to address the extent to which clinicians have a responsibility to review it all in an integrated EHR with many sources. Alternate venues for provider-patient correspondence, such as emails and EHR messages, create additional healthcare encounters, increased documentation burdens, and liability risks [31]. Anesthesiologists and other clinicians may be predisposed to information overload, leading physicians and other providers to overlook key findings despite reliable access to documentation [30]. The clinician who misses a critical detail that affects treatment decisions may be liable for negligence; this is true even when a detail is missed at a different institution [30]. Additionally, the institution in which the clinician works can also be considered liable under the respondeat superior doctrine, where an employer is held accountable for the actions of their employees [30]. 

### Metadata and Audit Trails

EHRs may also increase clinicians’ responsibility and accountability [30, 32]. An audit log, also known as an audit trail or event log, is a chronological record of activities in an EHR system. An audit log tracks who accessed the system, when, from where, and what they did. This includes logging in, entering or modifying data, viewing parts of the chart, and printing [33]. This is metadata – data about the data – that is captured in a log in nearly all EHR systems. Metadata describes actions that are performed on EHR data. This includes who logged into an EHR, what records they requested to view when data were entered, and other information associated with entries in the record, including the edit history. There are multiple strategies used by EHRs to record data about events and the relationships between them [34]. This metadata is not visible to the casual users of these systems and creates an audit trail that can be seen and used in malpractice cases.

Thus, an EHR audit log creates the opportunity to answer certain questions: Has the information presented by the EHR been changed after it was captured? This includes physiologic data, mechanical data (e.g., fresh gas flow rates) and the content of written notes. An audit log will also include the time of day that a user logged on and when they were presented with a note. Did a clinician see an important note immediately, or hours after it was written? Did they write their progress note at the time stamped on it, or did they write it hours earlier or later, and change the timestamp? As this metadata is not typically described as part of the medical record, and is not, as yet, routinely printed when charts are sent from one institution to another (or as part of discovery in a lawsuit), logs that answer these questions may pose significant risk, or quality improvement opportunities, to an institution or group of clinicians. Sophisticated electronic auditing procedures can identify individuals who reviewed, or failed to review, key information.30 Documentation templates that automatically import information may introduce new liabilities; information copied and pasted from other clinicians’ notes may contain inaccurate or incomplete information.30.

The term'discovery'refers to the activity conducted before a trial to find facts and evidence [35]. The procedural rules of discovery have been broadened in the pretrial period to prevent surprise evidence at trial. Every keystroke leaves a footprint, and although the audit trail information may not be included in the patient’s legal medical record, it is nonetheless discoverable. This discoverability underscores the potential impact of audit logs on legal proceedings. For example, consider note completion's timeliness after a critical incident. In a case of medical negligence, the defense might argue that the delay in charting was due to the need for a physician to care for the patient. From a plaintiff’s perspective, that same delay might signal the desire to craft the note to mitigate liability risks more carefully.

### Metadata Discoverability

When a medical malpractice case arises, the EHR and audit logs help determine which individuals and health organizations were involved in a patient’s care. Such knowledge can be used to guide risk management, attorneys, and expert witnesses with issues around prosecution and defense [36]. ^[ii]^While audit logs may assist in medico-legal activities, limitations also exist. An audit log does not fully account for the entire clinical activity in question. Though EHR audit logs are a legal requirement, not all EHR vendors track and store the same information with the same degree of detail. Some healthcare workflows are complex, and an audit log may lack enough detail to characterize an activity fully. Audit logs have also been shown to have varying degrees of inaccuracy [37]. In a medico-legal dispute, the hierarchy of evidence considers the “chart” supreme. The court considers the narrative account of involved parties and will listen to court-recognized experts when necessary. However, the modern chart, or EHR, will be viewed as the most compelling datapoint. The fidelity of the EHR is critical, and the legal requirement to protect patient confidentiality by an audit log may still generate insufficient legal coverage in accusations of negligence.

**Fig. 2:**
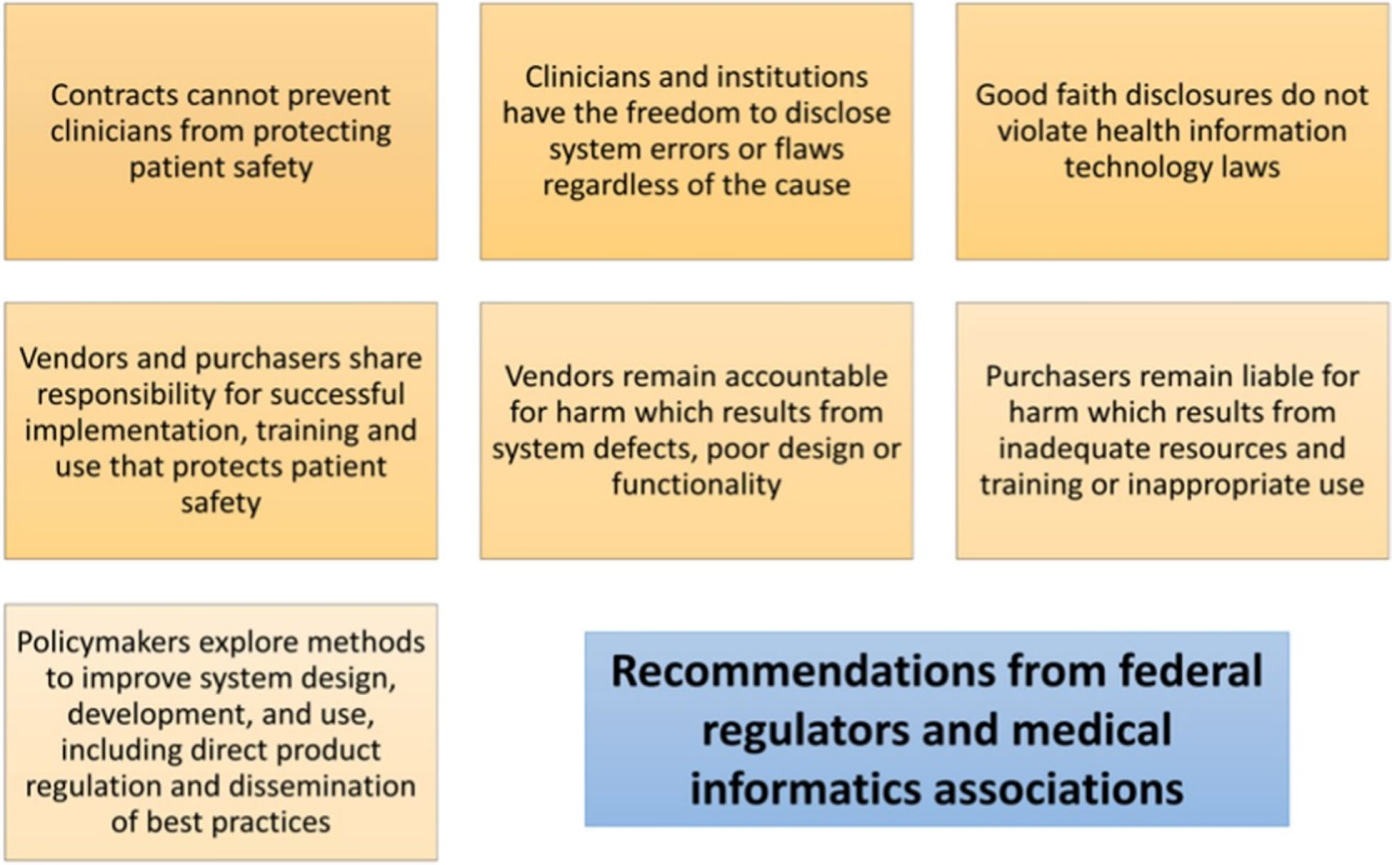
Recommendations for EHR use provided by federal regulation and medical informatics associations

In addition to subpoenaing the medical record, underlying metadata may also be subpoenaed for use in a malpractice lawsuit. Metadata may be used to suggest physician misconduct, misdiagnosis, and lack of credibility and vigilance. In large and complex systems with documents collected from several institutions and health information exchanges, the likelihood that relevant information may be overlooked is much greater than when the chart contains limited data. Additionally, scanned documents may be burdensome and difficult to read, which may cause delayed review of large charts [38]. Overrides of CDS alerts or reminders and clinician departure from guidelines and recommendations leave an electronic footprint that can lead to liability in the case of an adverse event [27]. This data can bolster the case for the plaintiff [38]. Federal regulations include notable provisions regarding the management of EHR, which include recommendations and rules that encourage improved functionality (Fig. 2) [39]. It is thus important for an institution to be aware of what logging is available on their systems.

Legal liabilities associated with EHR systems are unclear. Many EHR contracts include a “hold harmless” clause which restricts the ability of clinicians to report EHR-related problems. The U.S. Office of the National Coordinator for Health Information Technology in 2013 released a guide to EHR contracts that may be used to guide purchasers of this software [40]. The report details commonly used instances of indemnification against EHR developers for claims brought regarding personal injury, death, privacy or security violations or intellectual property infringement. This language often shifts responsibility to the purchaser/user regardless of the cause, if the claim arises from the use of EHR. The U.S. FDA has requested healthcare organizations to report EHR-related safety hazards, enabling processes to do so [30]. Genomic data and predictive analytics will likely lead to heightened privacy and insurability concerns along with new ethical and legal dilemmas [39]. 

The standards for the use of electronic information in litigation are rapidly changing and the rules of electronic discovery (e-discovery) are emerging and evolving. Physician workarounds may result in inconsistent data documentation practices which pose potential medicolegal risk by increasing the chances that key patient information could be outdated and overlooked by other users [41]. Ethical concerns may also be present with EHR use, such as determining who has ownership of protected health information and who informs patients of privacy breaches. 

Before the widespread adoption of the automated anesthesia record, individual anesthesiologists exercised discretion on the nature of recorded information. These actions were predicated on a belief about what was an expected physiologic response to various stimuli. As a result, hemodynamic records were, at best, a much more qualitative record and at worst, a work of fiction. In anesthesia jargon, “train tracks” described a record with very little moment to moment hemodynamic variation [42]. In the paper record era, such a document was created and advanced as evidence for a well conducted anesthetic. In truth, hemodynamic parameters are stochastic, and a lack of variability is generally indicative of physiologic distress. A loss of heart rate variability has, for example, long been recognized as correlated with increased mortality after myocardial infarction [43]. 

In the lead up to the anesthesia record keeping transition from paper to electronic, the Anesthesia Patient Safety Foundation (APSF) raised a concern that the new record keeping system would increase the negligence litigation risk. This was based on the belief that when the automated anesthesia record displayed the expected much wider variability, any negative outcome would now be attributed to this new more variable hemodynamic finding [44]. Around that same time, the APSF also stated that an automated record would provide contemporaneous and unbiased information about intraoperative events and therefore may help refute any claims of negligence. The statement reasoned that an automated record would support a claim that intraoperative responses were appropriate and logical [45]. 

It is well established that in claims of negligence, poor record keeping is associated with a poor defense [46]. From the court’s perspective, post hoc charting or a spoken recall of the narrative events will be the least compelling evidence. The medical record is considered the gold standard of evidence, and more information is generally better than less. The risk is that an unvarnished and unbiased account might indeed point to negligent actions; nonetheless, medical practice is not benefitted by a culture designed to obfuscate objective data. The best automated anesthesia record not only captures all relevant information but can be designed to prompt and further document the standard of practice.

## Key Case Studies in EHR-Related Litigation

The use of EHR has been consequential in litigation associated with patient care, such as the case of Vargas V. Lee [47]. In 2012, Jose Vargas underwent surgery at Wyckoff Heights Medical Center to correct a congenital left clubfoot. Postoperatively, the patient developed gangrene in his foot, ultimately requiring amputation. He then sued for malpractice. As part of discovery, the hospital produced the plaintiff’s EHR. The plaintiff requested that the audit trail for these records also be produced, and the hospital objected.

The plaintiff’s stated reason for the request for the EHR metadata was to authenticate the medical records to ascertain whether “the patient records that were eventually provided to them were complete and unaltered”. They argued that given “the failure to timely and properly diagnose and treat plaintiff’s medical complications following surgery, the requested portion of the audit trail was relevant to the timing and substance of plaintiff’s care following surgery.”

If earlier records were deleted or rewritten, or if later notes were back timed to appear as if they were written contemporaneously with events that happened hours or days earlier, that knowledge could be used to disprove the veracity of the record and to support a case for negligence.

Although the medical center initially objected to metadata disclosure, the court noted that “plaintiff’s request was limited to the period immediately following the injured plaintiff’s surgery.” The court ruled that “the requested audit trail was relevant to the allegations of negligence that underlie this medical malpractice action in that the audit trail would provide, or was reasonably likely to lead to, information bearing directly on the post-operative care that was provided to the injured plaintiff.”

Therefore, the court ordered the hospital to supply the EHR metadata and audit trail to the plaintiff. The plaintiff prevailed in the final verdict of the lawsuit, though it is unclear how much the audit trail from the EHR contributed to this outcome. Nonetheless, that the court required the hospital to provide the metadata has long-lasting relevance.

Similar claims regarding the production of metadata occurred in Miller v. Sauberman. In this case, a New York Supreme Court Justice compelled the production of metadata related to the plaintiff’s medical records despite the defendant’s estimated cost of $250,000 to produce the records [48]. The medical record initially provided to the plaintiff showed conflicting entries on the same day regarding the presence or absence of pressure ulcers. The plaintiff wanted these datato determine the identity of the clinician who firstnoted the presence of such ulcers and when that occurred. In this instance, the court ruled that the “plaintiff is entitled to the metadata for his medical record to determine if the medical record was altered, and if so, when and by whom.” Although the hospital required the services of a third party to help generate this audit log, the fact that their EHR was outdated and poorly supported also hurt the case of the defendant.

In another legal case, Gilbert v. Highland Hospital, the audit trail was sought to establish if the attending physician had reviewed the plaintiff’s medical records prior to their discharge from the emergency department [49]. Metadata was sought to help in “quantifying the level of involvement of the emergency department attending physician with the decedent’s care.” The court agreed with the plaintiff’s request that they were entitled to the additional records. Generally, it has been found that the audit trail information is material to the case"if there is any possibility that the information is sought in good faith for possible use as evidence-in-chief or in rebuttal or for cross examination [49].” 

Requests for information from the audit trail can be broad in EHR-related litigation.In Baker v. Geisinger Cmty. Med. Ctr., the audit trail was sought for “any purpose (access, modification, etc.) by any person” [50]. The period of inquiry for the audit request also extended beyond the plaintiff’s discharge date from the hospitalization. To gain a more objective understanding of an event’s timeline, the review of audit information helped provide more insight into the presence or involvement of other health care providers who may not have been explicitly mentioned in prior medical notes [50]. As previously discussed, malpractice claims analyzed over a two-year period using the CRICO Comparative Benchmark System (CBS) found 248 cases in which EHR-related events were cited as contributing factors in the legal action, highlighting the broad ways in which the EHR can both contribute to care concerns as well as serve as a source for discovery [28]. 

## Best Practices for EHR Governance and Implementation 

An EHR transition team is a potential way to smoothly implement the new system, and it should be multidisciplinary. cohort of EHR “champions” and superusers are valuable to provide guidance during implementations and ongoing support for struggling clinicians. These individuals greatly facilitate initial implementation and proper documentation practices, although the champions’ definitive efficacy may often not be quantified via traditional investigatory methodology [51]. Providers should also be educated about the EHR’s capabilities, such as the ability to retrieve audit trails and archiving of changes to a patient’s medical record.

### Medicolegal Considerations, Risk Mitigation and Institutional Policies

There are essential considerations for anesthesiologists by which they should approach these medicolegal concerns with EHRs. Anesthesiologists should understand the most important EHR-related audit trail policies, procedures, education, monitoring and enforcement activities at their respective organizations [36]. One comprehensive review of “best practices” surrounding EHR audit logs includes the following recommendations [36]:

1. Establish a policy for how long after clinical or administrative events details can be charted (e.g., 24 h in for inpatients).
2. Identify and document which parts of the patient’s electronic record will be produced in response to a request from a plaintiff attorney for the “complete medical record”.
3. Review the EHR's “print patient record to file” functionality to make sure it matches the policy for what will be produced following a subpoena.
4. Identify a departmental super-user of the institution’s EHR who has enhanced privileges, including the ability to generate a patient’s complete electronic health record for quality and patient safety review, or for legal purposes.
5. Identify a departmental super-user who is enabled to generate audit trails for specific patients as needed for quality, patient safety and medicolegal review.

Procedures such as adherence to “log in/log out” best practices and minimizing customization from national standards to enable interoperability and data sharing are also crucial [52]. This often includes such practical steps as not sharing passwords and logging out when leaving or not at a computer terminal.

### Ethical and Regulatory Considerations

**Fig. 3:**
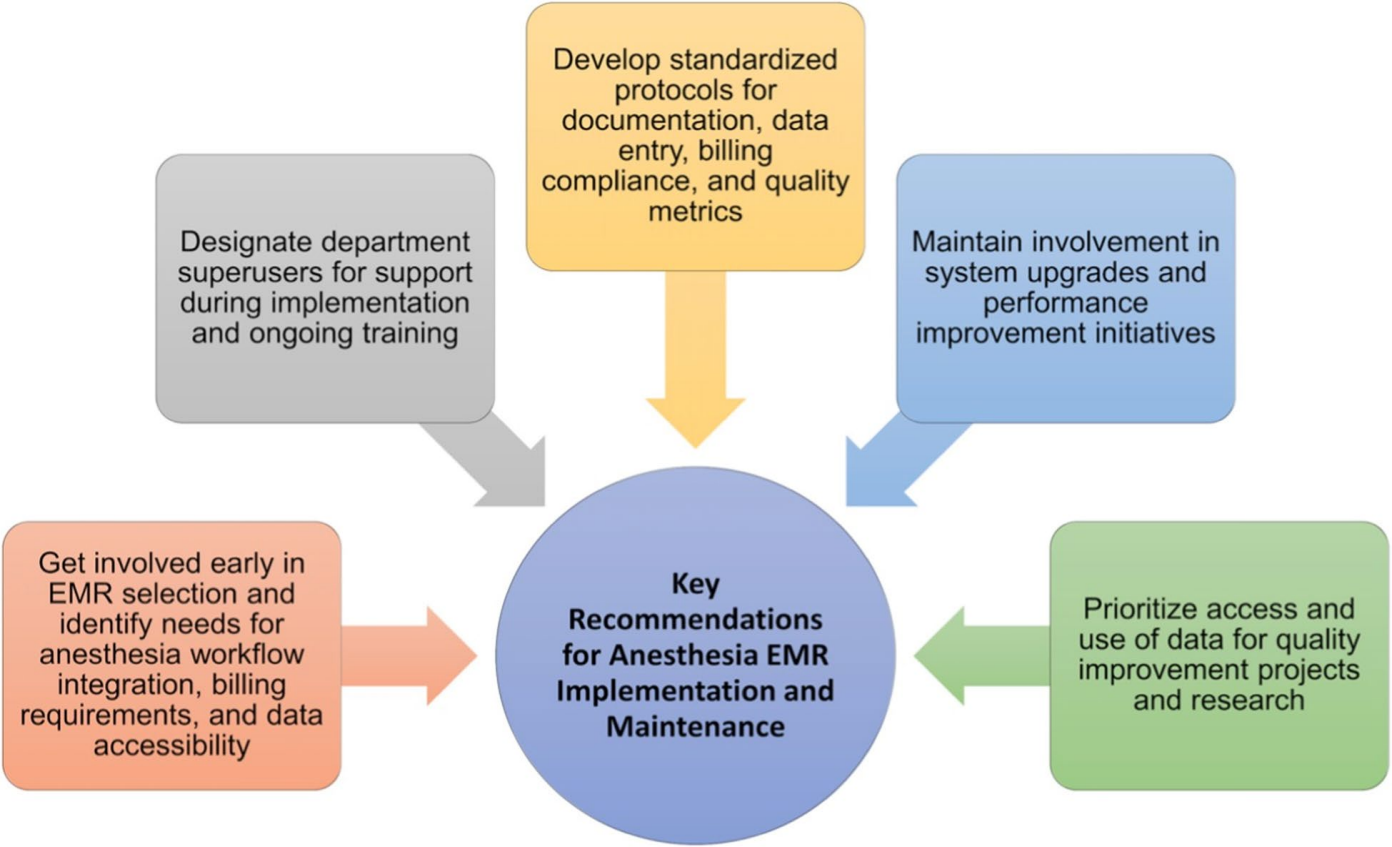
Key recommendations for anesthesia EHR implementation and maintenance

There are several recommended best practices for the anesthesia team to use EHRs effectively during anesthesia care (Fig. 3). It is important for anesthesiologists to be involved with hospital management and IT steering committees to ensure that anesthesiology-related interests are considered when choosing EHR software. There are several EHR system options offered for anesthesia and hospital systems. Oracle Cerner (Oracle Corporation, Austin, TX) and Epic (Epic Systems, Verona, WI) are two notable EHR systems featured in large medical centers. Scaled down EHRs for ambulatory centers with embedded anesthesia modules are available too. For smaller, scaled down systems, significant cost savings can occur with monthly billing and shorter installation periods [53].

 The Multicenter Perioperative Outcomes Group, which uses its integrated EHR to enhance quality, educational and research initiatives, has several recommendations to ensure data quality [5]. These include a requirement that data must be available at the local level before transfer to their coordinating center, rigorously validated against standardized metrics before use, curated into computable phenotypes that are easily accessible, and collected for both quality improvement and research purposes given their synergy [5]. 

Further, anesthesia groups should also consider the holding period for billing charges before submission to ensure timely completion of documentation [54]. Industry-wide improvement of anesthesia modules in EHR systems should include a movement toward standards for clinical terminology in anesthesia to allow for easier data management [55]. In anesthesia practice, common data exists such as ASA Physical Status classification, Mallampati score, and delineations of types of anesthesia techniques. However, in SNOMED, anesthesia-related terminology is sometimes ambiguous and duplicated. For example, postoperative nausea and vomiting is a common data point that is not yet extracted easily in many EHRs, possibly due to a lack of uniform methodology [55]. 

### Future Challenges: AI, Interoperability, and Cybersecurity

Cybersecurity risks should also be a major concern for practicing clinicians and anesthesiologists in particular. They should be mitigated according to hospital and practice policies. Recent trends in anesthesia including the frequent use of wireless systems, and integration of nearly every device with the internet have rendered this specialty at particular vulnerability to cyberattacks [56]. A review by Kruse et al. recommends that healthcare entities clearly define cybersecurity duties, ensure adequate policies for data breaches and software upgrades, utilize cloud-based computing and instruct users not to access suspicious code, amongst other suggestions to avoid systemic risk [57]. A successful mitigation strategy requires coordination amongst information technology, clinical and administrative leaders to ensure protection of relevant data including protected health information as well as maintaining digital infrastructure required for normal operations [58]. For example, the risk of ransomware is especially grave for healthcare institutions, and its prevention requires a coordinated, multidisciplinary approach. It requires education and training for frontline clinicians and users, it requires formalized policies and procedures for administrators, and requires active vigilance and monitoring from IT personnel. Healthcare facilities owe a duty to their patients to protect their data from breaches or potential manipulation that may result in suboptimal care or compromises of data for such nefarious ends as sale to malicious third parties.

Future integration of artificial intelligence and machine learning into EHR systems for anesthesia presents further opportunities to improve workflows and patient safety. Artificial intelligence may involve incorporation of patient data to produce real-time suggestions and warnings like current clinical decision support systems [59]. Anesthesiologists should endeavor to participate in the planning, evaluation and implementation of these systems in the perioperative setting with a solid understanding of the medicolegal risks and liability associated with their use. Further, it is important to consider and assess the role of AI and large language models in medicolegal risk. It will be important to determine whether or not the authors or owners of algorithm or models that are incorporated into patient care bear any liability when they contribute to aberrant medical care. If, as expected, AI continues to affect the doctor-patient relationship, by guiding the preoperative evaluation process, or playing a role in postoperative care, will the burden of blame shift away from clinicians to programmers and computer scientists? Large language models and AI may be used to summarize medical chart information and provide recommendations to clinicians; however, it remains to be determined who will assume liability if there are gaps in that information or errors in synthesis.

Interestingly, AI and large language models may also provide a potential solution for a medicolegal issue of the utmost importance and salience. That issue concerns timely sharing of electronic health data with patients secondary to the Cures Act, requiring timely access to EHR for patients. Time pressures and constraints may affect clinicians’ ability to provide data and information in the chart that accurately reflects the clinical care provided for patients. Herein, the careful and thoughtful integration of AI and LLMs into EHRs may satisfy the needs of patients to access their data in a timely manner, but also clinicians who want to ensure accuracy in the EHR of the narrative of patient care.

## Conclusion

Electronic medical records have revolutionized healthcare delivery over the past decade. They were adopted with the high hopes of improving quality of care & patient outcomes, reducing costs and facilitating business and administrative functions. Only some of these goals have been partially realized while new challenges, hazards, and liabilities emerged. Achieved benefits include legible note writing, remote access to clinical information and care delivered elsewhere, computerized order entry, improved workflow efficiency, billing capture and revenue cycle management. With further evolution, clinical decision support tools and best practice advisories are now embedded in the EMR. In addition, the accumulation of big data enables large-scale research and quality metrics measurement and reporting.

The improved clinical documentation and proper use of EMR may help defend a claim and decrease medicolegal liability. However, the EMR also has several disadvantages and can contribute to increased medico-legal risks. Liability may arise from patient harm attributable to poor inaccurate documentation, failure to review relevant information, even when care was rendered elsewhere many years prior. The common use of copy and paste functions propagates inaccurate and outdated information. Reviewing the huge amount of captured information is unrealistic and the extent of liability from not reviewing every detail is still unclear. Alarm fatigue and overrides can lead to liability if an adverse outcome occurs after an appropriate clinical decision support alert is ignored. Additional risks may arise from patient privacy (HIPPA) breaches and cybersecurity attacks that disrupt clinical operations and expose protected health information. Detailed logs of who accessed the record & when, along with revision history trails are tracked and stored. This metadata is discoverable during medicolegal claims. Altering the medical record after an event will likely damage defendants’ credibility. Institutions should follow best practice recommendations for EMR implementation, data security and training of clinicians and end-users. Big data collection requires a sophisticated infrastructure for digital connectivity and data warehousing along with robust data governance policies and procedures. Commercial artificial intelligence tools introduce new clinical, ethical and liability concerns requiring adequate regulatory oversight and vigilance with clinical adoption. The extent to which the EHR increases or decreases medicolegal liability in anesthesia practice remains to be determined.

## Supplementary Information

Below is the link to the electronic supplementary material.

Supplementary file1 (DOCX 17 KB)

## Data Availability

No datasets were generated or analysed during the current study.
