# Supplementary material for: Narrative Review of Electronic Health Record Systems in Anesthesia: Benefits, Risks, and Medico-Legal Considerations in the United States of America
Source: J Med Syst. 2025 Jun 23;49(1):87. doi: 10.1007/s10916-025-02221-z (PMC12183129; doi:10.1007/s10916-025-02221-z)
Supplement: Supplementary file 1 — Supplementary file1 (DOCX 17 KB) [file 10916_2025_2221_MOESM1_ESM.docx]

**Appendix A**

**Narrative review of electronic health record systems in anesthesia: benefits, risks, and medico-legal considerations in the United States of America**

**Contributors:**

***CIIT Collaborators:***

- Vikas O’Reilly-Shah MD, Associate Professor- Anesthesiology, University of Washington

Contributed to: research hypothesis, background research, drafting manuscript, editing manuscript

- Kent B. Berg, MD, MBA, Clinical Associate Professor, Vice Chair of Anesthesiology Information Systems, Thomas Jefferson University

Contributed to: research hypothesis, background research, drafting manuscript, editing manuscript

- Ellen Wang MD, Clinical Associate Professor of Pediatric Anesthesiology and Medical Director of Clinical Informatics, Stanford University

Contributed to: research hypothesis, background research, drafting manuscript, editing manuscript

- Jonathan M. Tan MD, Vice Chair (Interim) of Analytics and Clinical Effectiveness, Department of Anesthesiology Critical Care Medicine, Assistant Professor of Clinical Anesthesiology and Spatial Sciences, Keck School of Medicine of USC

Contributed to: research hypothesis, background research, drafting manuscript, editing manuscript

- Kristin Ondecko MD FASA, Assistant Professor Anesthesiology, University of Pittsburgh Medical Center

Contributed to: research hypothesis, background research, drafting manuscript, editing manuscript

- Mark Banoub MD, Staff Anesthesiologist, Director of Cardio-thoracic Anesthesia Director, Henry Ford Medical Center

Contributed to: research hypothesis, background research, drafting manuscript, editing manuscript

- Michael Pesce MD, Anesthesia Residency Program Director at HCA Healthcare, HCA Healthcare

Contributed to: research hypothesis, background research, drafting manuscript, editing manuscript

- Reem Khatib MD, Assistant Professor Anesthesiology, Cleveland Clinic

Contributed to: research hypothesis, background research, drafting manuscript, editing manuscript

- Ramon E. Abola MD, Associate Professor, Director of Communication, Deputy Chief Medical Information Officer, Stony Brook Medicine

Contributed to: research hypothesis, background research, drafting manuscript, editing manuscript
